# Negotiating the polypharmacy paradox: a video-reflexive ethnography study of polypharmacy and its practices in primary care

**DOI:** 10.1136/bmjqs-2022-014963

**Published:** 2022-09-02

**Authors:** Deborah Swinglehurst, Lucie Hogger, Nina Fudge

**Affiliations:** 1 Wolfson Institute of Population Health, Queen Mary University of London, London, UK; 2 QMUL

**Keywords:** Complexity, General practice, Medication safety, Quality improvement methodologies, Social sciences

## Abstract

**Background:**

Polypharmacy is an important safety concern. Medication reviews are recommended for patients affected by polypharmacy, but little is known about how they are conducted, nor how clinicians make sense of them. We used video-reflexive ethnography (VRE) to: illuminate how reviews are conducted; elicit professional dialogue and concerns about polypharmacy; invite new transferable understandings of polypharmacy and its management.

**Methods:**

We conducted 422 hours of fieldwork (participant observation), filmed 18 consultations between clinicians and patients receiving 10 or more regular items of medication (so-called ‘higher risk’ polypharmacy) and played short clips of film footage to 34 participants (general practitioners, nurses, clinical pharmacists, practice managers) in seven audio-recorded reflexive workshops. Our analysis focused on ‘moments of potentiation’ and traced clinicians’ shifting understandings of their practices.

**Results:**

Participants rarely referenced biomedical aspects of prescribing (eg, drug-drug interactions, ‘Numbers Needed to Treat/Harm’) focussing instead on polypharmacy as an emotional and relational challenge. Clinicians initially denigrated their medication review work as mundane. Through VRE they reframed their work as complex, identifying polypharmacy as a delicate matter to negotiate. In patients with multimorbidity and polypharmacy it was difficult to disentangle medication review from other aspects of patients’ medical care. Such conditions of complexity presented clinicians with competing professional obligations which were difficult to reconcile. Medication review was identified as an ongoing process, rather than a discrete ‘one-off’ activity. Meaningful progress towards tackling polypharmacy was only possible through small, incremental, carefully supported changes in which both patient and clinician negotiated a sharing of responsibility, best supported by continuity of care.

**Conclusions:**

Supporting acceptable, feasible and meaningful progress towards addressing problematic polypharmacy may require shifts in how medication reviews are conceptualised. Responsible decision-making under conditions of such complexity and uncertainty depends crucially on the affective or emotional quality of the clinician-patient relationship.

**WHAT IS ALREADY KNOWN ON THIS TOPIC**

- Polypharmacy is a global safety concern.
- Regular medication reviews are recommended but little is known about how reviews are conducted in practice.

**WHAT THIS STUDY ADDS**

- Problematic polypharmacy is not just a technical problem but a relational challenge.
- Medication review is not a ‘one-off’ activity, but an ongoing collaborative process characterised by small, incremental changes.
- Responsible decision-making under conditions of complexity and uncertainty calls for an ethical-affective mode of decision-making that arises from the quality of the clinician-patient relationship.

**HOW THIS STUDY MIGHT AFFECT RESEARCH, PRACTICE OR POLICY**

- Using video-reflexive ethnography enabled new transferable understandings of polypharmacy and medication review which challenge key policy assumptions about how to tackle polypharmacy.

## Introduction

Tackling polypharmacy is a global safety issue.^1^ Older patients affected by polypharmacy warrant particular attention.^2 3^ Increasing age and increasing numbers of medications both contribute to escalating risks,^4–6^ with 10+ medications regarded as ‘higher risk’ polypharmacy.^7^ Regular medication reviews are strongly recommended for older patients experiencing polypharmacy^8–10^ although it remains unclear whether these result in clinically significant improvement.^11 12^ Despite the limited evidence base, concern about the scale and potential impact of polypharmacy has culminated in the National Health Service (NHS) England Network Contract Directly Enhanced Service including a requirement that patients prescribed 10 or more medications are invited to a structured medication review (SMR).^13–15^

The National Institute of Health and Care Excellence defines medication review as a ‘*structured, critical examination of a person’s medicines with the objective of reaching an agreement with the person about treatment, optimising the impact of medicines, minimising the number of medication-related problems and reducing waste’.* 16 Most key policy and professional documents take care to ensure that medication review is *not* framed primarily as a mechanism to reduce medications; polypharmacy is sometimes ‘appropriate’.^7 17 18^

Few studies have focused on *how* medication reviews are conducted in practice.^19^ In the context of older people experiencing polypharmacy this is fraught with complexity. Single disease guidelines advise when to start new drugs but seldom address when to stop them. They are informed by population-based evidence derived from randomised controlled trials which often exclude older people with multimorbidity. Ageing and multimorbidity thus drive an accumulation of medicines and increasing therapeutic uncertainty, as clinicians weigh up unmeasurable harm against benefit. Furthermore, clinicians are incentivised through initiatives such as the Quality and Outcomes Framework to manage disease and disease risk according to the ‘single disease’ model, while at the same time being encouraged to address the harms of problematic polypharmacy which may emerge directly from their efforts at ‘quality’.^15 20^

Polypharmacy is a ‘wicked problem’. Wicked problems are characterised by several intractable difficulties. These include: difficulties *defining* problems (what distinguishes an observed condition from a desired condition?); difficulties *locating* problems (where in the complex causal networks does the trouble really lie?); and difficulties identifying the actions that might narrow the gap between *what-is* and *what-ought-to-be* (the boundary between ‘appropriate’ and ‘problematic’ polypharmacy is fuzzy).^21 22^

General practitioners (GPs) are considered well placed to manage polypharmacy,^23^ but they face a complex, unsettling and inherently paradoxical scenario, caught in a situation of irreducible uncertainty.^24^ Furthermore, they are just one part of a system-wide network of people, practices, norms and conditions that together sustain polypharmacy. Addressing problematic polypharmacy is likely to require system-wide change. This raises unanswered questions such as what do GPs *do* when faced with this ‘polypharmacy paradox’ and how do they make sense of their activities?

We report findings of a video-reflexive ethnography (VRE) study. Our aims were threefold, to: illuminate how medication reviews are conducted in practice in the context of polypharmacy; elicit professional concerns about polypharmacy practices; harness the potential of VRE to provoke new transferable understandings relevant to the process of managing polypharmacy.

## Methods

### VRE as catalyst to change

VRE unearths ‘taken-for-granted’ habituated activities of practice that have often become invisible, even to the actors involved.^25^ It involves filming people ‘in situ’ doing their work, showing short clips to relevant actors and inviting them to view their familiar practices from an unfamiliar vantage point. The clips are a catalyst for reflexive interprofessional deliberation. They encourage people to: ‘see differently’ everyday practices which might otherwise remain invisible; appreciate the complexity of their work; identify possibilities for change. VRE has been deployed across many healthcare activities including infection control practices in hospitals,^26^ explorations of how the built environment supports safe communication in intensive care units,^27^ and studies of leadership development.^28^

VRE is based on four principles: *exnovation* (foregrounds the accomplishment and complexity of care practices and assumes opportunity for ‘innovation-from-within’); *collaboration* (participatory co-creation); *reflexivity* (participants review and reimagine their practices); *care* (creates a safe space for participants).^25^ Iedema describes VRE as a ‘potentiation technology’. Potentiation indicates *movement*, in the sense of both *understanding* and *affect*. VRE enables participants to:

- Orient to paradox/contradiction
- Loosen attachments to existing positions, arrangements, meanings, selves, structures, practices
- Explore alternatives to what is ‘taken-as given’ and experiment with expectations of the future.^29^

Our VRE study was part of a wider ethnographic project investigating polypharmacy and multimorbidity in primary care (*Addressing the Polypharmacy Challenge in Older People with Multimorbidity, APOLLO-MM*).^30^ We recruited three NHS GP practices, four community pharmacies, 24 patients and engaged in longitudinal ethnographic observation spanning patients’ homes, general practice and community pharmacy. Full details of our methods are published elsewhere.^30^

Table 1 sets out our approach to VRE. Table 2 shows brief details of the research sites and participation in the VRE workshops.

**Table 1:** Study design and approach using VRE

Process/stage	Procedures	Dates
Ethnographic observation	DS (academic GP/social scientist), NF (social anthropologist) both experienced ethnographers in health settings and LH (speech and language therapist undertaking an ethnographic PhD) spent 422 hours observing practices relating to medicines management across the three GP sites and recruited a cohort of 24 patients, aged 65+ who were prescribed 10 or more items of medication (site A n=7, site B n=10, site C n=7). Our general practice observations enabled us to shadow staff and follow organisational routines relating to medicines. We followed patient participants ethnographically for a period of 18–24 months observing their lived experiences of multimorbidity, polypharmacy and healthcare services.	September 17 to March 20
Filming medication reviews/consultations where medications were discussed	DS, LH, NF accompanied study patients to 29 selected clinical consultations when we anticipated discussion of patients’ medicines. NF and LH video-recorded 18 consultations. In addition to consent to taking part in the wider APOLLO-MM Study, all participants (patients and practitioners) involved in video-recorded consultations engaged in a further consent process before and after the consultation ensuring full understanding of how video data would be used. 11 practitioners (site A n=2, site B n=3, site C n=6) and 11 patients (site A n=3; site B n=3; site C n=5) took part in 18 video-recorded consultations. The duration of the video-recorded consultations ranged from 5 to 32 min (mean=17 min).	June 8 to February 20
Video analysis and selecting critical moments	DS and NF viewed each video-recording independently multiple times, identifying ‘critical moments’, then met to discuss and refine our selection as we prepared video clips to share with practitioners in VRE workshops. Each researcher drew up tables identifying sections of potential interest in the footage, a brief description, and a rationale for selecting this footage as a ‘critical moment’. We did not strictly define what might constitute a ‘critical moment’ *a priori*, but this iterative process brought together: Our research interest in how practices ‘on the ground’ constitute, sustain or challenge polypharmacy Insights derived from our ethnographic observations of patients and practitioners about how polypharmacy is ‘done’ Moments of communicative interest (eg, misunderstandings; the evolution and emergence of decisions; occasions when the direction of the consultation appeared to ‘turn’) ‘professional noticing’^38^—moments in which clinicians exercised their professional vision in action (eg, selecting, interpreting and responding to relevant events to support patient care) Footage that might extend or illuminate insights that emerged through professional dialogue in previous VRE workshops This was an iterative process, occurring alongside ongoing data collection and delivery of VRE workshops. Clips were selected for their potential to invite discussion regarding ‘ordinary’ practice.	November 18 to February 20
Reflexive workshops	This was a flexible and dynamic process, each 1 hour VRE workshop contributing not only to our evolving understanding of polypharmacy and its practices, but also to our understanding of *how* to maximise the potential of the VRE methodology to *realise* the complexity of polypharmacy and stimulate practitioners to engage in productive dialogue across our three settings (eg, we discovered that the shortest video clips—even those of 11 s—were often most effective at supporting reflexivity). We often played clips several times (guided by participants) and used different clips in each workshop. We provided written prompts to stimulate discussion, which participants used to varying degrees: What’s happening in this clip? What do you notice? Does anything surprise you? How do you feel when you watch this footage? Is there something that this clip does NOT show that is important for understanding what is going on? VRE workshops were audio-recorded and transcribed.	June 19 to March 20
Participant reflection and evaluation of VRE workshops	At the end of the third (final) workshop at sites A and B we spent 15 min inviting the participants’ reflections on the VRE approach. Participants also completed a short written reflective activity identifying the most important thing they had learnt from taking part and something they would consider doing differently as a result of the VRE workshops.	February 20 to March 20
Analysis of VRE transcripts	After repeated reading and familiarisation we identified ‘moments of potentiation’ and traced how these played out within workshops. Our analysis was iterative, enabling us to tailor our selection of video clips to deepen our insights as workshops progressed. Once all workshops were complete we conducted further rounds of analysis, honing in on shifts in how polypharmacy and medication review practices were articulated and understood by participants through their participation in the VRE process, identifying key themes across the data set.	June 19 to October 21

APOLLO-MM, Addressing the Polypharmacy Challenge in Older People with Multimorbidity; GP, general practitioner; VRE, video-reflexive ethnography.

**Table 2:** Details of research sites and participants

	Site A	Site B	Site C
Brief description of GP sites A, B and C (at recruitment)	Urban practice c.11000 patients, in second most deprived decile*	Suburban practice, c.13000 patients, in least deprived decile	Urban practice, c.14000 patients in most deprived decile
Number of patients recruited from each site	7	10	7
Number of videos	5	4	8
Critical moments identified	13	16	10
Number of reflexive workshops	3	3	1†
Participants in the VRE workshops	12	13	9
General practitioner	9	9	6
Practice nurse	1	1	3
Clinical pharmacist	1	2	–
Practice manager	1	1	–

*Details from National General Practice Profiles produced by Public Health England Data Science).

†Only one workshop held at site C due to the COVID-19 pandemic.

GP, general practitioner; VRE, video-reflexive ethnography.

### PPI statement

We have a project advisory group with 11 members: lay chair; academics; health professionals; representation from Age UK; 2 patient members. We have an online patient panel of five members. Patients were involved in: proposal development; designing participant materials/project website (www.apollosocialscience.org); application for ethical approval; project launch event; piloting interviews; study design and conduct.

## Results

Medication reviews were conducted regularly in all three practices and documented with a formal code in patients’ electronic records (‘*medication review done’*). They were an important aspect of repeat prescribing routines. Receptionists did not issue repeat prescriptions to patients with ‘overdue’ review dates, instead alerting a GP or clinical pharmacist to take action. Clinicians sometimes advanced the date in patients’ records based on a notes review and sometimes invited patients to book a medication review appointment. Reviews were usually face-to-face with a GP (towards the end of our fieldwork some were conducted by newly employed clinical pharmacists). Reviews often exceeded the scheduled 10 min (only 3 of 18 filmed consultations were under 10 min; 7 exceeded 20 min). Clinicians also undertook brief opportunistic ‘medication reviews’ within consultations booked for other reasons.

VRE workshops surfaced professional concerns that we had not encountered elsewhere in our research since polypharmacy was rarely discussed in everyday practice. Although participants oriented to polypharmacy as a complex and challenging ‘problem’ they did not use the term ‘problematic polypharmacy’ explicitly. An overarching finding was that participants rarely referred to the biomedical aspects of prescribing which typically dominate professional texts and tools for prescribing safely (eg, the evidence supporting prescriptions, drug-drug interactions, adverse drug reactions). Rather, they foregrounded emotional and relational aspects of their activities, the challenges of managing time and their perplexity at how to respond to seemingly incommensurable professional obligations.

### ‘Just a medication review’—appreciating the complexity within the mundane

Clinicians typically responded to the first clip they viewed by denigrating their work as mundane (‘*it’s just a medication review*’; ‘*I was just reviewing as usual’*). They explained how dedicated medication reviews often became entangled with other concerns (‘*it always gets squeezed in’; ‘it gets crowded out’; ‘it’s the stuff that gets pinched really’*) and were prone to being trumped by ‘*more pressing problems’*. As they spent more time watching footage and discussing their observations they re-articulated their practices:

People are trying to do like a super-complex bit of work within 10 to 15 minutes [GP7 Site C workshop 1]

It just made me realise how complex the whole polypharmacy is. There’s so many different factors, both for me and for the patients. And actually, I’m someone who likes to think systematically about things, and it’s very difficult to think systematically about something that is this complex… just an awareness of that is helpful.’ [GP1 Site B workshop 3].

We’re fighting a losing battle sometimes. I mean…he’s not unusual in his complexity (murmurs of agreement) A lot of our patients are equally complex and we are trying to do too much in our consultations (loud murmurs of agreement) [GP2 Site A workshop 1]

We now illuminate this complexity further by focussing on two aspects of medication review that VRE participants grappled with across sites. First, we consider how clinicians organised their consultations, and how VRE discussions led them to reconceptualise medication review as ‘ongoing activity’ rather than discrete event. We then show how VRE potentiated a shift in participants’ understanding of ‘responsible’ decision-making as they discussed tackling polypharmacy through de-prescribing (stopping medicines).

### Organising the medication review

Although several formal tools exist to support SMRs (eg, STOPP/START, NO TEARS) our participants did not use these in practice nor refer to them in VRE workshops.^31 32^ Participants acknowledged that until the VRE workshops they had not thought critically about how they organise their medication reviews at all.

#### The computer as organising tool

In Site B we showed a clip of a clinical pharmacist looking at the computer and remarking “*Paroxetine is the first medication on there*”. A GP commented:

It’s interesting. I don’t, I don’t think I even think about how I introduce going through people’s meds consciously. So that, I can see that, that, that I have no, in my head, I probably have no structure in mind in doing a meds review. [GP1 Site B workshop 2]

As participants brought this tacit work of organising into the realm of conscious deliberation, they recognised that medication reviews were *‘computer heavy’*, or even an opportunity to ‘*tidy up the record*’. The computer tended to encourage them to simply work ‘*down the list*’ of medications one by one (‘*it was just top of her list’*; ‘*it’s just because when you look at the screen it’s on the top*). Electronic records could be configured to list medications in different ways: alphabetically or by date of addition to the ‘repeats’ list. Most participants did not know how lists were configured on their own computers and had not previously thought about how and to what extent this shaped their organisation of reviews. In each site there was at least one participant able to explain how their own IT systems were configured and how they could be reconfigured:

Y’know you notice on the list there’ll be, y’know’ whatever, in whatever order. But it’s nothing to do with the drug, it’s about when it was issued last [GP7 Site C workshop 1]

It’s much more helpful to have them in chronological order…I seem to remember it got changed and I was very pleased when it was changed back because that was really unhelpful [GP3 Site C workshop1]

You can order them alphabetically, which is least helpful obviously, or you can order them in the, at the, in date (order) when they were added which is the most helpful…you do pick up quite a lot from how long they’ve been on what, don’t you, not even necessarily consciously…I don’t think I’ve thought about it until it got raised, and now I’m thinking about it…I flick my eyes, I probably flick my eyes down, like you do, to see if nothing’s been used at all, or if it’s been underused or overused [GP1 Site B workshop 2]

#### Introducing polypharmacy to the medication review: a delicate matter

The opening of the medication review was a key moment in its organisation. In Site C, we showed a clip of a GP turning to the computer, raising his voice in pitch and volume and exclaiming: *‘OK, so in terms of your medication I can see you are on quite a lot of medication aren’t you?’* This clip prompted lively discussion about: how to embark on reviews; whether and how to draw attention to polypharmacy as a focus of concern; how to mediate between computer and patient in ‘*computer heavy*’ consultations.

For example, in the following response a GP gently cautioned against suggesting a patient’s polypharmacy may be ‘*too much*’ and invited his colleagues to consider a more ‘*neutral*’ stance. This quote (and the clip which prompted it) frame polypharmacy as a delicate matter to negotiate:

I was going to say about the clip y’know (name of GP) used a particular way of introducing the subject and I’m wondering if I do have a kind of standard way of actually kind of bringing it up y’know. As people pointed out, the suggestion is, from what you said, that you’re on too much almost, aren’t you…I wonder if there’s a less sort of, just a more neutral way of bringing up the subject [GP11 Site C workshop 1]

One GP explained she often pivots the medication screen to share it with her patients, hence mitigating the problem that *‘you’ve been dragged to the screen by medication*’ and simultaneously avoiding judgemental statements about polypharmacy: *‘Oh, shall we have a look at what medicines you’re on?’* Later in the same workshop we showed a video of a different GP engaging in this screen-sharing. This invited praise from one colleague:

I like how (name of GP) turned the screen towards the patient, you know the sort of joint assessment, almost like the ball is in your court [GP5 Site C workshop 1]

Another was more guarded:

It’s difficult though, isn’t it, because I think if you’ve got a full medication screen its sort of inviting somebody in to look at something that they don’t understand, because you need such high literacy skills…and our patients know their drugs by a different name…sometimes I I w- wonder actually what are you sharing when you invite somebody to look at it? [GP7 Site C workshop 1]

We now present an alternative approach to opening and organising the consultation.

#### The medicines as tools for organising

In Site A, patients were encouraged to bring their medicines to review appointments. Participants watched a clip in which the GP began by asking the patient if she had brought her medicines. The woman, who was in her late 60s, slowly took her medicines, one by one, from her bag with her arthritic hands and laid them carefully on the GP’s desk. She then got up from her chair, walked slowly to her shopping trolley to fetch her inhalers, then sat down, breathless.

The GP shown in the clip initially evaluated this sequence by describing it as ‘*haphazard*’ but on further reflection it prompted him to:

“wonder what her expectations from the medication review might be” adding “as they see something it kind of triggers something else: “yeah I might need some more of those, and can you…?" So it’s just trying to organise those tasks" [GP1 Site A workshop 1]

This clip, and the GP’s appraisal of it, offered his colleagues a way of re-imagining the organising process: ‘*trying to organise*’ the *‘tasks’* of the medication review. He points to: putting the patient’s expectations first; the medicine boxes offering material prompts to elicit the patient’s concerns; an intention to organise the review in a way that responds to the patient’s predicament rather than the computer list. His colleagues voiced admiration at the GP’s apparently calm, unhurried, ‘patient-focused’ approach, yet were concerned at how time-consuming this was (*‘I mean that took her five min to get her medication ou*t!’). Loosened from the attachment to the computer as key organiser of work, they wrestled with two seemingly incommensurable professional obligations—patient-centred practice and efficiency, a tension which previous research has highlighted.^33^

In the next section we show participants reconceptualising the medication review, in a move that goes some way to addressing this aspect of the polypharmacy paradox.

#### Medication review: from discrete event to ongoing activity with fuzzy boundaries

Participating in VRE led participants to shift from regarding medication reviews as discrete events towards a realisation that although *documented* as discrete ‘completed’ units of activity, polypharmacy reviews necessarily stretched across several consultations and remained incomplete with no clearly defined beginning or end. Participants regarded it ‘*impossible’* to conduct a review within the allocated time and constructed the notional ‘10 min appointment’ as illusory. Organising medication reviews could only be understood alongside wider contexts of organising.

It was difficult to disentangle medication from patients’ wider health concerns. In this quote, one doctor—a GP trainer—explained how she clusters medicines mentally enabling a ‘problem oriented’ approach rather than a medicines-driven approach, dealing with a limited number of problems over a series of consultations:

I’m trying to encourage us to review the problems, rather than call it a med review. So you bring up osteoporosis and you then review the medications, the Dexa scan, then you review your coronary heart disease and is the blood pressure done, and are there any blood tests to do? And do you need to change your medication? So you’re reviewing each problem and you might deal with two and then bring them back for the other four, or whatever. I’m trying to encourage that [GP9 Site B workshop 1]

Participants concluded that successfully balancing the paradoxical demands to be both patient-centred and efficient within a busy clinic rested entirely on ‘*knowing the patient’* and ‘*know(ing) how long they’ve been on tablets for*’. It meant resisting what they regarded as an increasing problem in general practice of clinicians conducting complex consultations with patients unknown to them:

I think it’s unsafe. If you’ve got ten minutes and they’ve got ten different problems over ten medications, how can you possibly get yourself up to speed? [GP7 Site A workshop 1]

Some clinicians wanted to limit the burden of repeat attendances for older patients with complex needs, and saw this as a justifiable reason for longer consultations, although it meant running late:

With this sort of female I’d just make everybody else wait, I would do more to try and at least make a tiny dent in the burden of coming back…but I- I- I- you can’t…that is a tiny dent isn’t it in her overall stuff? [GP1 Site B workshop 2]

Running late was not the only way in which complex medication reviews spilled over into unscheduled time. Participants also discussed the work they do *outside* the consultation, delving into patients’ extensive records to work out why medicines had been prescribed.

Engaging in VRE led participants to recognise that meaningful progress towards tackling polypharmacy was only possible through small, incremental, carefully supported changes (*‘one thing at a time’, ‘slow and steady’, ‘focus on one thing you can achieve’*). This is a useful point of departure for our next theme.

### Responsible decision-making on changing and stopping medicines

Workshop discussions often centred on concerns around who is responsible for decision-making in the context of polypharmacy. Participants recognised that decisions about reducing or stopping medicines were complex and potentially involved ‘*upsetting the status quo’,* acknowledging that ‘*it’s often an awful lot easier just to maintain medication than alter it’*. As well as the uncertain risk of ‘*destabilising*’ someone, there was the additional discomfort of stopping medicines prescribed by other clinicians:

It raises the question about who addresses the issues. So he isn’t your…you’re not the main GP [GP5 Site C workshop 1]

The more medication, the less likely you are to engage. If I have a very complex patient who I don’t know – it’s a person in my head who has been seen by the nephrologist, they’re on buckets of drugs. In fact it’s sort of almost a red flag for getting involved if they’re not my patients, because, you know, you have to have experience of why they’ve accumulated all these pills (murmurs of agreement) [GP7 Site C workshop 1]

One newly appointed clinical pharmacist who had previous experience in an NHS hospital pointed out that she had encountered similar concerns from secondary care clinicians (‘*Well, actually the GP knows the patient, we’ll let the GP review it and the GP will stop those.’*).

We illustrate this theme by detailing the conceptual shifts that emerged in our final workshop at Site A, thus highlighting more clearly how the VRE methodology encourages a loosening of attachments to existing positions and exploration of alternatives; enabling the emergence of new, transferable understandings.

We shared two clips in this workshop. The first clip (see box 1) showed a GP explaining to a patient: ‘*there’s probably nothing I can stop in terms of your heart tablets’*. The second clip (see box 2) showed a short extract in which patient and GP negotiate a reduction in furosemide (a medicine which some study participants referred to as a ‘heart tablet’). The first clip illustrated a phenomenon we encountered across our data set, that ‘heart tablets’ prompted particular displays of resistance to change. The second was one of several examples of a patient inviting their GP to consider reducing a medication. Sometimes these invitations were overlooked. In this example the GP takes up the patient’s suggestion. We were curious to know what might emerge in the VRE workshop by juxtaposing these two clips.

Box 1 **An account of shifting notions of sharing responsibility**

Clip 1 (11 seconds): The general practitioner (GP) looked at his computer, highlighted several drugs on the screen, then turned to the patient: *‘From the point of view, I think, of stopping anything today, there’s probably nothing I can stop in terms of your heart tablets’.*

The GP in the clip (Dr Rose) explained that he was looking at different groups of medications with a view to ‘*rationalising medications*’ (‘*this patient is always keen to stop any medications that aren’t indicated*’). In the discussion that followed a GP colleague (Dr Green) added: ‘*I think, we would feel probably a little bit disempowered changing that, because it is started by the hospital…we don’t have a lot of control*’. Participants discussed the special difficulty of reviewing and changing medications prescribed by others (‘*I never feel I’m in a position to alter them’*).

Dr Green went on: ‘*I wonder if it’s easier to contact the consultant and say ‘Is it OK? …I do that occasionally’*‘ but a colleague pointed out that often the patient has been discharged from consultant care some time ago and questioned ‘*How confident do they feel…do they hit back and say ‘actually you now know more than me about the story, you need to make that decision’’*.

Another clinician added:

*‘I would get advice from cardiology, speak to them…just run it past them, speak to the on-call cardiologist…just run it past them…that would reassure me’*. A discussion ensued about different ways of seeking specialist advice, until Dr Rose reflected:

‘*If you think about it, you’re calling an on-call cardiology registrar who might be ST4 or ST5, or just really quite new in the job and because they’ve got this title of being the specialist you’re taking their words as authority…as generalists why don’t we feel the confidence to…(murmurs of agreement). Do we need assent from them*?’

This reflection ushered in a ‘turn’ in the discussion towards a recognition that while seeking advice from the specialist enabled them to document *‘spoke to cardiologist’* in the medical record, an alternative approach would be to seek a second opinion from a GP colleague within the practice. A highly experienced GP reflected on the role of medication in older patients: ‘*the benefits (of medication) get less and less and less’* and invited his colleagues to consider ‘*What is actually going to benefit them?*’ He went on to add ‘*So having the backing and giving us the authority back again to actually do that* (change or stop medications) *would be, for me personally, very helpful*’ a move which seemed to suggest that current conditions of practice made this difficult. Later in the workshop the same GP referred to ‘*too many dangers: political, social, GMC* [(General Medical Council])*’*.

DS invited participants to consider who might be the appropriate specialist for patients affected by polypharmacy. This prompted two responses: the patient (‘*sometimes patients stop it themselves’*) and the GP (‘*I mean we’re certainly expert in the side effects of all these things’*). A brief discussion ensued about the paucity of evidence to support clinicians (‘*most studies don’t apply to polypharmacy patients (murmurs of agreement) so we have to use our own clinical judgments and we should be using our own clinical judgement’*) and recognition of the dual challenge—or paradoxical tangle—that ‘*There are no simple answers. Every situation is different. It’s an individual patient situation*’ and ‘*the big problem is for the last 10 years we’ve been financially incentivised to put people on polypharmacy*’.

Box 2 **An account of negotiating de-prescribing**

Clip 2 (53 seconds):

General practitioner (GP): ‘*I suggest we leave the furosemide as it is until next week…until after you’ve had your echo*’

Patient: ‘*what about wh- should I perhaps reduce it?*’ … ‘*try to reduce it…see what happens*’ (he suggests he half the tablet down a ‘crack’ in the middle of the tablet)

GP: *you could try…see if it has an effect on your legs and if you notice a change to this noise you’re hearing, so there’s no harm in doing that*.

Patient: *I’ll try that*

Participants began by reflecting on the patient being ‘*in control’* of his pills, ‘*testing his own theory’* and the possibility that he wanted to stop furosemide completely, cutting it by half being a reasonable compromise, a ‘*negotiation’*—a ‘*middle ground’*. This moved into discussion about the ‘*weight of responsibility*’ for decision-making with one GP saying they ‘*wished*’ more patients would *‘take control’* like this, another saying they ‘*wished*’ more patients would make decisions so that responsibility for decision-making didn’t rest entirely with the GP. This prompted one GP to share a personal story about an illness experience in which they found it surprising to discover that they didn’t want to take on all responsibility as a patient.

Participants drew contrasts between different scenarios (eg, prescribing hormone replacement therapy, prescribing antidepressants) and considered how decision-making is ‘shared’ under different conditions.

Returning to Clip 2, one GP concluded ‘*when it’s a shared responsibility it reduces the burden a bit’*. His colleagues agreed.

DS and NF invited participants to reflect on points of similarity and difference with the previous clip (seebox 1) in which they had described seeking reassurance from a specialist. This led participants to conclude that a decision shared with a patient offered valid reassurance and— like their discussions with consultants—was a form of shared responsibility that they could document in their records. In concluding reflections on the workshop one GP summarised this discussion:

*‘That little insight…of sharing responsibility with the patients actually as being equivalent to the consultant. Very interesting. I found that fascinating. Actually I might try to work that in there. I might try to encourage* it’.

Together these extracts show VRE as an intervention capable of prompting profound shifts in how practitioners understand and value their role and their relationship to others within the wider system (eg, consultants, specialists, colleagues, patients). Moreover, the process generated valuable insights regarding the potential that *already exists* within the therapeutic encounter and within generalists’ capabilities to tackle complex decision-making under conditions of uncertainty. Responsible, incremental change to tackle polypharmacy *is* within reach and can be approached in ways that are acceptable to both clinician and patient.

## Discussion

In summary, our use of VRE methodology prompted new, transferable understandings regarding the process of managing polypharmacy. Polypharmacy was framed primarily as a relational, emotional concern rather than a biomedical one—a delicate matter that is difficult to negotiate. The ‘mundane’ medication review was reframed as ‘super-complex’ work, with computers and medicines boxes identified as organisers of this work. Our findings suggest that in the context of polypharmacy, medication review is not a ‘discrete activity’ (‘*medication review done*’) but is stretched across time and space and surprisingly difficult to disentangle from other aspects of medical care. It is an ongoing, incomplete, open-ended process characterised by tentative, small, incremental changes enacted through what Mol may refer to as ‘tinkering’^34^ and most safely approached in the context of relational continuity. Polypharmacy presents practitioners not only with a problem of ‘lack of evidence’ to guide actions, but with incommensurable professional obligations. This, combined with concerns about responsibility and accountability in the face of inescapable uncertainty may fuel professional disempowerment and inertia.

If policy efforts to implement SMRs for complex, high-risk patients are to succeed in effecting meaningful improvement to patient care, then considerable effort will need to be invested in ensuring that the entanglements between medicines and wider health concerns can be addressed simultaneously. Isolating ‘medicines’ for review may be impossible. Efforts to ‘structure’ or organise the review must ensure that patients can contribute to how meaningful organisation is achieved. Inviting patients to bring their medicines to consultations may help communication and enable patients to identify and prioritise their concerns. Efforts to adjust medications amidst this complexity are likely to require ongoing opportunities for follow-up; ‘one-off’ reviews may miss the mark.

Our research offers optimism that clinicians are both willing and capable of rising to these challenges, using resources that are already available to them within the therapeutic encounter. However, ensuring success may require both continuity of care, and professional opportunities for reflexive peer-to-peer support (such as our participants experienced through VRE).

Reflecting on their VRE experience, several participants identified transformation in their learning and new areas of capability:^35^

I suppose for me it would be about making it less medication-centred and trying to think about that patient and what it might be like for them. It’s not something that comes naturally I think, as you’re running late and trying to whizz through everything. I think that maybe trying to just stop and say ‘How would it be for them on these many, many different medications? What might make it a bit easier? What might make things better for them or improve their life?’ [GP7 Site B workshop 3]

That decision-making might shift from being a decision *about the patient* (by inviting specialist expertise or the reassurance of a GP colleague) towards a *dialogue with the patient* (boxes 1 and 2) represents a profound shift. This is not simply an appreciation of patients’ ‘expertise’, rather a movement in the notion of accountability and a reworking of the clinician-patient relationship. We witness a shift away from the patient being regarded as an object of concern towards appreciation of the patient as a unique being, situated within a specific context. This finding resonates with Sellar’s account of ‘responsible uncertainty’.^36^ Drawing on Bauman,^37^ Sellar points to an ethical-affective level of responsible decision—a highly situated mode of ethical decision-making that derives from *being-for* the other (ie, not simply *knowing about* them). Under this lens, deliberative judgement, though important, is insufficient to discharge one’s ethical responsibility. Ethical responsibility also arises from the affective or emotional quality of the intersubjective relationship (eg, between clinician and patient).^36^ This has important implications. It suggests that responsible decision-making under conditions of complexity and uncertainty cannot be easily codified and may not translate readily to instrumental tools. Arguably this may explain why we did not encounter such tools in use. It also highlights the centrality of continuity of relational care as a means of enabling ethical responsibility.

A key strength of our work is that VRE’s dual investigative and interventionist approach allowed us to (1) Illuminate the polypharmacy paradox and (2) Identify shortcomings in ‘taken-for-granted’ notions of the medication review as a response to this paradox. Collectively participants reimagined innovative ways forward that better embraced the complexity they face. Importantly, this was a facilitated process. The *potentiation* unleashed by VRE applies to both research participants and researchers; it is through collaborative working that progress is made. As researchers, we selected clips. Workshops may have played out differently had we selected different clips, or if the participating clinicians had selected clips. A limitation is that the COVID-19 pandemic halted our research; we were unable to evaluate how participants integrated their learning into everyday practice. However, we achieved our key objective, namely to employ VRE methodology to bring about *potentiation,* generating new transferable understandings of the polypharmacy paradox relevant to both policy and practice.

## Data Availability

No data are available. Our ethics approval and consent procedures were based on the anonymity of the individuals who participated, hence further access to the full data set cannot be granted.
